# Algorithm based patient care protocol to optimize patient care and inpatient stay in head and neck free flap patients

**DOI:** 10.1186/s40463-015-0090-6

**Published:** 2015-11-02

**Authors:** Daniel A. O’Connell, Brittany Barber, Max F. Klein, Jeff Soparlo, Hani Al-Marzouki, Jeffrey R. Harris, Hadi Seikaly

**Affiliations:** Division of Otolaryngology-Head and Neck Surgery, Department of Surgery, University of Alberta, Edmonton, AB Canada; Faculty of Medicine and Dentistry, University of Alberta Hospital, 1E4.31 8440 112th Street NW, Edmonton, AB T6R 2B7 Canada

**Keywords:** Head and neck free flap, Patient care protocol, Inpatient stay, Algorithm based, Length of stay

## Abstract

**Objective:**

To determine if rigid adherence (where medically appropriate) to an algorithm/checklist-based patient care pathway can reduce the duration of hospitalization and complication rates in patients undergoing head and neck reconstruction with free tissue transfer.

**Methods:**

Study design was a retrospective case-control study of patients undergoing major head and neck cancer resections and reconstruction at a tertiary referral centre. The intervention was rigid adherence to a pre-existing care pathway including flow algorithms and multidisciplinary checklists incorporated into patient charting and care orders. 157 patients were enrolled prospectively and were compared to 99 patients in a historical cohort. Patient charts were reviewed and information related to the patient, procedure, and post-operative course was extracted. The two groups were compared for number of major and minor complications (using the Clavien-Dindo system) and length of stay in hospital.

**Results:**

Comparing pre- and post-intervention groups, no significant difference was identified in duration of hospital stay (21.5 days vs. 20.5 days, *p* = 0.750), the rate of major complications was significantly higher in the pre-intervention cohort (25.3 % vs. 14.0 %, *p* = 0.031), the rate of minor complications was not significantly higher (34.3 % vs 30.8 %, *p* = 0.610).

**Conclusion:**

Rigid adherence to our patient care pathway, and improved charting techniques including flow algorithms and multidisciplinary checklists has improved patient care by showing a significant reduction in the rate of major complications.

**Electronic supplementary material:**

The online version of this article (doi:10.1186/s40463-015-0090-6) contains supplementary material, which is available to authorized users.

## Introduction

Free flap reconstruction is a procedure performed by many head and neck surgeons to restore the form and function of the complex anatomy of the head and neck region following cancer extirpation. Due to the complexity of these reconstructive procedures, the likelihood of post-operative complications, the long length of stay in hospital, and the rigorous multidisciplinary team involved, head and neck cancer surgery is resource intensive, and as a result, is one of the most expensive solid malignant tumors to treat [1].

The economic burden of this disease has sparked much research into methods for effective management of resources and cost minimization. Patient care algorithms and care pathways have been shown to decrease complication rates and reduce length of stay post-operatively [2–4]. Care algorithms have been implemented and shown to be effective for post-operative patients for which the costs of care are high and for whom inefficiencies are likely to arise during their post-operative care. These care protocols are geared towards quality improvement in patient care, and intended to coordinate the ideal sequence and timing of staff actions for achieving patient goals with optimal efficiency [5–7].

An important feature of care pathways is their relationship to the patient record. The care pathway often replaces other documentation entirely (e.g. physician orders and progress notes), to reduce time spent by care providers completing paperwork. Patients that deviate from the pathway are flagged, information is collected about the deviation, and a plan to return the patient to the path is defined.Progress notes and record keeping by all allied health professionals is standardized and simplified [8, 9].

Over the past 20 years, many care pathways in medicine have been developed that were eventually disregarded despite their early success [7]. The biggest challenge to successful implementation of algorithm-based care pathways is the creation of a permanent medical record in a format that will be accepted and used by physicians. Standardized order sets and progress notes have been suggested as providing the best chance at physician acceptance [10, 11]. Creating standardized documentation that allows for effective analysis of deviations from the algorithm is another significant challenge [12]. The intention at our center is to create an evolving patient care algorithm that is subject to critical thinking and quality analysis. At our center, a patient care algorithm has been in place for many years. Patient care pathways, and various allied health professional goals and intervention time points were available in a standardized document for review, however were not directly incorporated into the patient record, potentially facilitating deviations from the patient care pathway for non-medically required reasons. Acknowledging this, the goal of our study was to determine if rigid adherence (where medically appropriate) to an algorithm/checklist-based patient care pathway could reduce the duration of hospitalization and decrease complication rates in patients undergoing head and neck reconstruction with free tissue transfer.

## Methods

This is a retrospective case–control study comprised of a historical control cohort, and a prospective sequential cohort. Both arms were enrolled in an algorithm-based care pathway designed specifically for patients undergoing free tissue transfer reconstruction. The intervention in the prospective cohort was rigid adherence to this care pathway using new flow algorithms and multidisciplinary checklists incorporated into patient charting and patient care orders (see Additional files 1 and 2) as well as requiring clinicians to record reasons and justifications for deviations from the established care pathway and facilitating analysis of deviations for possible appropriate interventions. The retrospective cohort followed the previous standard of care at our institution utilizing standard progress note formats (Subjective Objective Assessment and Plan) in post-operative charting as well as relying on individual assessments of clinicians in making the decision to implement the suggested time sensitive interventions of our critical care pathway.

The current care protocols at our institution were developed the format of the Gantt chart, having a time-task table linking components of care to a time-line. These protocols were generated with multi-disciplinary assessment, identifying and incorporating: a) best practices; b) defining the expected duration of hospital stays; c) a multidisciplinary analysis of the care process aimed at coordination to decrease time spent on the rate limiting steps; d) development of care maps to help all hospital staff understand their various roles in the care protocol; e) data collection as a quality control measure for identifying when and why patients deviate from the care pathway; f) decreasing nursing and physician documentation burdens; g) educating patients and their families about the goals of care to involve them more fully, hence improving patient satisfaction [7].

The new checklist charting format included a standardized patient intake form (Additional file 1) that provides all allied health professionals with a standardized description of the surgical procedure to ensure accurate baseline information for subsequent post-operative assessments. This was designed to reduce subjective charting and standardize nomenclature and descriptions of patient status and findings with standardized checklist progress notes being used to possibly improve efficiency and knowledge transfer during patient charting (Additional files 2 and 3).

To ensure that this study was appropriately powered, in-patient stay duration data from 125 consecutive patients previously treated with free tissue transfer was collected and analyzed. A sample size of 92 patients in each cohort would provide 80 % power to detect a reduction in in patient stay by 3 days at the α = 0.05 level. Although any reduction in in-patient stay can be associated with a reduced care cost and a clinically significant reduction in overall stay, the 3 day reduction was chosen to see if a larger cost reduction and statistically significant reduction could be confirmed. Patients were included in the study who had a free tissue transfer reconstruction of head and neck defects following cancer extirpation. Patients under 18 years of age, or whose hospital charts were incomplete or not available for review from our health records department were excluded. In addition, for patients whose hospital stay was prolonged due to lack of access to long-term care facilities alone, the date they were placed on the list for long-term care was recorded in our database as the date of discharge. Patients whose care deviated from the algorithm set forth by the care pathway were not excluded from the study. Data was collected prospectively for the intervention group and was obtained through a review of patient hospital, surgeon office and cancer centre charts for the retrospective control group. Information related to the patient, procedure, and post-operative course was extracted into a standardized data collection form for both cohorts. Retrospective data was collected independently by two separate reviewers and compared for accuracy. Any descrepencies in retrospective data was investigaed and clarified by a third reviewer. Length of stay was calculated from the date of surgery to the date of discharge or being listed for transfer to a long-term care facility.

Complications during in-patient stay were classified according to the Clavien-Dindo classificiation [13]. In this classification scheme surgical complications are graded 1–5 based on severity or amount of intervention required (see Table 1). Grade 5 complications relate to the death of a patient, grade 4 include life-threatening complications including any requiring ICU management, grade 3 complications require surgical, radiologic or endoscopic intervention (for the purposes of this study included need for second surgery, reconstructions, etc.) grade 2 are any complications causing a deviation from the normal post-operative course and requiring major pharmaceutical interventions, and grade 1 complications include any deviations from the post-operative course not requiring pharmaceutical or procedural intervention. Major complications were defined as Clavien-Dindo grades 3-5, while minor complications were defined as Clavien-Dindo grades 1-2.

**Table 1:** Classification of surgical complications based on the Clavien – Dindo system [13]

Grade	Definition
1	Any deviation from the normal post-op course without the need for pharmacologic treatment or radiographic, endoscopic or surgical intervention. Allowed interventions include antiemetics, antipyretics, analgesics, diuretics, electrolytes, and physiotherapy. Wound infections dealt with at bedside also included.
2	Requiring pharmogologic treatment with drugs other than such allowed for grade 1 complications. Blood transfusions and total parenteral nutrition included.
3	Requiring surgical, endoscopic or radiographic intervention
4	Life-threatening complications( including central nervous system complications) requiring ICU management
5	Death of a patient

The Chi squared test and student’s t-tests were used to compare patient demographics in both groups for categorical and continuous variables respectively. Logistic regression was used to analyze length of stay of patients with different classes of complications based on the Clavien-Dindo system. All statistical analyses were performed using Stata 12.1 (StataCorp LP, College Station, TX).

## Results

256 patients were included in our study, 99 patients in the control group and 157 in the intervention group. Details on patient demographics, baseline characteristics and co-morbities can be found in Table 2. There were no significant differences in these factors between the two patient populations (*p* > 0.05). Rates of tracheostomy in the two groups showed no significant difference (*p* = 0.977) Table 3. There was no significant difference between the intervention and control groups in mean length of hospital stay (21.5 days, vs. 20.5 days, *p* = 0.750). For the purposes of comparison the rates of complications were divided into major (Clavien-Dindo class ≥3) and minor (Clavien-Dindo ≤ 3). In the historic control group rates of major and minor complications were 34.3 % and 25.3 %, while those in the intervention group were 30.8 % and 13.7 %. Comparison between rates of minor complications between the two groups showed no significant difference (*p* = 0.610) while there was a significant difference in major complications between the two groups (*p* = 0.031), Table 4. The length of stay for patients who underwent microvascular free tissue transfer at different points of the week was also compared. A longer average length of stay was observed for those having flaps performed on days where critical intervention time points occurred on non-regular working days compared to other days. Specifically at our center, free flaps done on Tuesday and Wednesday were found to have an increased LOS compared to other days (*p* < 0.001).

**Table 2:** Comparison of patient demographics of the historical control and prospective cohorts

Parameter	Control	Intervention	*P*-value
Number	99	157	-
Age (years)	58.1	61.2	0.874
Gender			
Male	63	116	0.094
Female	36	41	
TNM Stage			
Stage I/II	18	19	0.194
Stage III/IV	81	148	
Comorbidities			
Tobacco user	77	108	0.151
ETOH	75	101	0.072
DM	9	18	0.677
CAD	12	9	0.100
Liver Disease	6	7	0.572
PVD	7	4	0.113
Dementia	0	0	1.000
CHF	1	2	0.847

*EtOH* History of regular alcohol consumption, *DM* Diabetes mellitus, *CAD* Coronary artery disease, *PVD* Peripheral vascular disease, *CHF* Congestive heart failure

**Table 3:** Rates of patients undergoing tracheostomy for airway management following major head and neck reconstruction

Airway	Historical	HNCCCP	*P*-value
Tracheostomy	84	133	0.977
No Tracheostomy	15	24

**Table 4:** Rates of major (Clavien-Dindo >3) and minor (Clavien-Dindo < 3) complications in patients undergoing major head and neck reconstruction

	Control group	Intervention group	*P*-value
Minor Complications (Clavien < 3)	34/99 (34.3 %)	48/157 (30.8 %)	0.610
Major Complications (Clavien ≥ 3)	25/99 (25.3 %)	22/157 (14.0 %)	0.031

## Discussion

Post-operative complications following HNCS have been shown to significantly increase hospital stay durations, mortality rates and the costs associated with care. The fact that post-operative care pathways following HNCS have the ability to reduce complication rates, in-patient stay duration and reducing the costs associated with treatment have been shown by multiple studies in North America, including a recent Canadian study confirming the cost-efficacy of care pathways in a publically funded health-care system [4].

The study presented was not designed as a validation study to examine the utility of patient care pathways in the HNCS population but rather to see if further improvements can be made in patient care when the use of care pathways is reinforced with enhanced patient records, and multidisciplinary checklists. The new checklist charting format included a standardized patient intake form (Additional file 1) that provides all allied health professionals with a standardized description of the surgical procedure to ensure accurate baseline information for subsequent post-operative assessments. We believe that this enhanced the information provided to all health professionals who came into contact with the patients following their HNCS. Standardized intake forms allows all allied health professionals to quickly adapt their intervention strategies based on each patient’s particular treatment related needs. Also standardized checklist progress notes were instituted. Although not directly measured we believe that this improved efficiency and knowledge transfer during patient charting (Additional file 2). Guiding the clinicians to justify various potential deviations from the care pathway, as well as reinforcing daily assessments of different aspects of healing and recovery during each HNCS patients in-patient stay.

We believe that the reduced complication rates identified in the study could be a direct result of earlier detection of pending complications facilitated by the enhanced patient record as well as providing reinforcement for critical thinking when faced with clinical findings or scenarios that might lead to various major and minor complications.

Our intervention of an algorithm/checklist based care path to reinforce the use of our pre-existing care pathway, has been shown reduce the rates of major and minor complications in patients undergoing microvascular free flap reconstruction of the head and neck. We have also shown a statistically significant correlation between the presence of major complications. One study demonstrated that post-surgical complications resulted in a 70 % + increase in true costs. The same study demonstrated that post-surgical complications following microsurgical reconstruction for head and neck cancer statistically significantly increased length of stay [14]. Although we were not able to demonstrate an overt statistically significant decrease in length of stay in this study, there was an observed measurable reduction in overall duration of inpatient stay (reduction of 1 day in the intervention group compared to the historical cohort). This overall reduction in in-patient days although not reaching statistical significance may be clinically significant in terms of its economic impact. The overall reduction in length of stay by 1 day in the intervention cohort translated into an elimination of 157 unessential in-patient days for our patient population. Based on current health region rates of $2780 (Canadian) per day on an advanced surgical ward this translates into a possible $436,460 Canadian dollars in savings to the healthcare system. As our study was powered to detect a mean difference of 3 inpatient days between the two test groups, further continued studies using larger cohorts may show further differences in overall length of stay following our intervention.

The type of free flap used in microvascular reconstruction plays an important role in the postoperative course of patients, as do the defect being reconstructed, indication for surgery and patient co-morbidities. These variables were not analyzed in our study, and are acknowledged study limitations. The lack of blinding and randomization are also limitations to this study however are inherent in a case–control design.

Care pathways are heavily relied upon in some areas of medicine and provide merely a guide in others. This study aimed to evaluate the effectiveness of specific adjuncts to our particular care pathway in improving patient care. The promise of cost reduction while maintaining or improving quality of care is often cited to support the use of care pathways. Implicit in this statement is that care pathways must be referenced and used to aid in patient care. However the fact that care pathways and algorithms are dynamic and subject to physician decision-making and autonomy must be acknowledged and understood when attempting to apply them to complex patient care environments.

Our study shows that adjuncts such as enhancing patient records and charting to enable the utilization and reinforcement of patient care pathways, as well as providing a mechanism for collecting data surrounding specific instances where the care pathway was not followed, can have the effect of reducing major complications rates following HNCS. Future directions of this research include an in-depth analysis of deviations from the pathway and further development of the document in efforts to maximize patient outcomes and minimize health care costs.

## Conclusions

Facilitation of the use of an algorithm based patient care pathway in head and neck cancer reconstruction via enhanced patient charting, and multidisciplinary checklists, has translated into a statistically significant reduction in the rates of major post-operative complications in patients undergoing major head and neck reconstruction at our institution. Rigid adherence where medically appropriate to an established patient care pathway following head and neck cancer reconstruction enhances patient care by reducing overall rates of major surgery-related complications.

## Additional files

Additional file 1:
**Head and neck oncology clinical care pathway.** Revised clinical care pathway. (DOC 115 kb) Additional file 2:
**Head and neck reconstruction flow sheet.** Daily progress note template. (PDF 1004 kb) Additional file 3:
**Head and neck reconstruction intake form.** Intake form for head and neck reconstruction patients. (PDF 1015 kb)

## Abbreviations

HNCS: Head and neck cancer surgery
LOS: Length of stay
POD: Post-operative day
OR: Operating room
ICU: Intensive care unit
