# Supplementary material for: Algorithm based patient care protocol to optimize patient care and inpatient stay in head and neck free flap patients
Source: J Otolaryngol Head Neck Surg. 2015 Nov 2;44:45. doi: 10.1186/s40463-015-0090-6 (PMC4631082; doi:10.1186/s40463-015-0090-6)
Supplement: Additional file 1: — Head and neck oncology clinical care pathway. Revised clinical care pathway. (DOC 115 kb) [file 40463_2015_90_MOESM1_ESM.doc]

Patient Label

***Clinical Care Pathway: Head & Neck Resection and Free Flap Reconstruction***

INITIAL CONSULTATION AND WORK-UP OF THE HEAD & NECK PATIENT

- History and Physical
- CT Scan of the Neck with IV Contrast
- MRI if any suspected skull base or prevertebral involvement
- CT Scan of Chest
- PET/CT Scan^1^
- Adjunct investigations
  - Laboratory: CBC, Electrolytes, Creatinine, Urea, INR, PTT, ALT, AST, GGT, Total Bilirubin, Type and Screen
  - ECG if age > 50 or cardiac history
- Quadroscopy and Biospy
  - On-call E24 if in-patient
  - Outpatient procedure to be arranged by surgeons office
  - Patient to follow-up with Head & Neck Surgeon 2 weeks post-operatively to review pathology

1. PET/CT and CT Neck can be done in single radiology appointment if requested

BIOPSY PROVEN HEAD & NECK CANCER

- Patient reviewed in Head & Neck Surgeon’s clinic to discuss biopsy and pathology results
- Patient referred to Head & Neck Cancer Clinic
- Patient reviewed by multi-disciplinary team at Head & Neck Cancer Clinic
- Treatment Pathway Selected by Patient
  - Primary Surgical Resection/Reconstruction – adjuvant Radiotherapy +/- Chemotherapy
  - Primary Radiotherapy +/- Chemotherapy

Patient Label

***Clinical Care Pathway: Head & Neck Resection and Free Flap Reconstruction***

SURGICAL WORK-UP AND PLANNING:

- Analyze tumour primary site and decide on type of resection and reconstructive options
- Location of Primary Tumour: ______________________
- Suspected Defect Description: _____________________
- Neck Dissections Planned: Yes □ No □
  - Right: Levels I □ II □ III □ IV □ V □
  - Left: Levels I □ II □ III □ IV □ V □
- Type of Reconstruction Confirmed
- If Free Flap Reconstruction:

Free Flap: ___________________ Side: Right □ Left □

- If Radial Forearm Free flap:
  - Patient handedness: Right □ Left □
  - Allen’s Test: Adequate Ulnar Collateral Flow: Yes □ No □
- If Fibular Free Flap or Posterior Tibial Free Flap:
  - Arrange Angiographic studies of lower limbs
- Surgical and Post-operative rehabilitation counseling performed by Surgeon(s) and/or Advanced Practitioner
- Pre-Admission Clinic Consultation
- Anesthesia Consultation
- ICU Consultation
- Medicine Consultation (if multiple medical co-morbidities)
- All pre-operative investigations performed and results reviewed
- Active Blood Type and Screen available on day of surgery
- Distant Metastasis definitively ruled out

Patient Label

***Clinical Care Pathway: Head & Neck Resection and Free Flap Reconstruction***

**POST-OP DAY 0 Date:**

**Patient Care**

- Check Physician’s Orders
- Patient transferred to ICU
- Initial flap assessment performed with resident on arrival to unit
- Flap checks q1h: colour; capillary refill; temperature; and Doppler
- All flap checks findings to be recorded in patient records
- 1:1 Nursing care
- Vital signs and Input/Output q1h
- Pain assessment q1h
- **If oral cavity/oropharynx surgery, no oral suction**
- Head of Bed > 30 with head in neutral position
- Assess for neck symmetry and swelling q1h
- Patient repositioning q2h
- Assess JP drain output q4h, reprime JP bulbs q4h and prn
- Tracheostomy cuff to remain inflated
- Ventilatory assistance as needed
- Re-enforce dressings as needed
  - Flap donor site
  - Skin graft donor site
- Feeding tube secured to nose
- No tube feeds
- IV fluids as ordered
- Electrolyte replenishment protocol filled out if not Renally impaired AND no other precluding pathology.

**Teaching / Discharge Planning**

- Reinforce use of analgesic
- Ensure nursing staff are comfortable with flap location and flap checks
- ***NO ORAL SUCTION***
- Contact family to discuss patient condition and operative findings
- Instruct family members on how to best communicate with patient and provide alternate forms of communication for patient if patient unable to speak

Patient Label

***Clinical Care Pathway: Head & Neck Resection and Free Flap Reconstruction***

**POST-OP DAY 1 Date:**

**PATIENT CARE**

- Close Observation Nursing
- **If oral cavity/oropharynx surgery, no oral suction**
- Monitor vital signs q4h and prn
- Trach cuff inflated
- Wean off ventilator to continuous cold neb via inline suction system
- Maintain SaO2 > 92%
- Chest assessment q4h and prn
- Assess need for tracheal instillations (NS) and suctioning q4h and prn
- Assess tracheal secretions for colour, consistency, volume, and odour
- Deep breathing encouragement
- Trach care with NS and polysporin ointment q4h
- Flap checks q1h. Record each assessment. Report any signs of decreased flap circulation to resident or staff physician immediately
- Pain assessment q1h
- At each shift change, incoming and outgoing RN to perform combined flap assessment. Both sign assessment form in patient record
- Head of Bed > 30 with head in neutral position
- Encourage patient to swallow own secretions
- Assess for neck edema and symmetry
- Neck incision line care q8h with NS followed by polysporin ointment
- Assess JP output q4h. Reprime and drain q4h and prn
- Reinforce flap donor and skin graft site dressings prn
- Gentle mouth care with NS q4h and prn
- Foley to urometer. Record intake / output q1h
- Check feeding tube placement qshift
- Ankle flexion and extension exercise q1h

**NUTRITION**

- NPO
- IV as ordered
- Start tube feeds as ordered. If any nausea or vomiting, reconsult dietician
- Once tube feeds started flush 250cc H20 via feeding tube q4h

**CONSULTS**

- Physiotherapy
- Dietician
- Respiratory therapy

**ACTIVITY**

- Turn and reposition q2h with head neutral
- Activity as tolerated – up to chair bid
- Thigh free flap – WBAT (no knee flexion)

**TEACHING/DISHCARGE PLANNING**

- Reinforce use of analgesic
- Reinforce importance of humidification with trach cradle
- Instruct family members on how to best communicate with patient and provide alternate forms of communication for patient if patient unable to speak
- ***No oral suction*** (potentially damages flap and promotes reliance on suction to clear oral secretions)
- Teach importance of swallowing oral secretions (retrains muscles altered by surgery)

Patient Label

***Clinical Care Pathway: Head & Neck Resection and Free Flap Reconstruction***

**POST-OP DAY 2 Date:**

**PATIENT CARE**

- Close Observation Nursing
- **If oral cavity/oropharynx surgery, no oral suction**
- System assessment qshift and prn
- Monitor vital signs q4h and prn
- Trach cuff deflated
- Continuous cold neb
- Maintain SaO2 > 92%
- Chest assessment q4h and prn
- Assess need for tracheal instillations (NS) and suctioning q4h and prn
- Assess tracheal secretions for colour, consistency, volume, and odour
- Deep breathing encouragement
- Trach care with NS and polysporin ointment q4h
- Flap checks q2h. Record each assessment. Report any signs of decreased flap circulation to resident or staff physician immediately
- Pain assessment q2h
- At each shift change, incoming and outgoing RN to perform combined flap assessment. Both sign assessment form in patient record
- Head of Bed > 30 with head in neutral position
- Encourage patient to swallow own secretions
- Assess for neck edema and symmetry
- Neck incision line care q8h with NS followed by polysporin ointment
- Assess JP output q4h. Reprime and drain q4h and prn
- Reinforce flap donor and skin graft site dressings prn
- Gentle mouth care with NS q4h and prn
- D/C Foley.
- Accurate ins and outs q4h
- Check feeding tube placement qshift
- Ankle flexion and extension exercise q1h during waking hours

**NUTRITION**

- IV TKVO when TF initiated.
- Start tube feeds as ordered. If any nausea or vomiting, reconsult dietician
- Once tube feeds started flush 250cc H_2_0 via feeding tube q4h

**CONSULTS**

Following:

- Physiotherapy
- Dietician
- Respiratory therapy
- Occupational Therapy to make splint for RFFF/FFF

**ACTIVITY**

- Turn and reposition q2h with head neutral
- Activity as tolerated – up to chair bid
- Fibular and ALT free flap – WBAT (no knee flexion until POD#7)

**TEACHING/DISHCARGE PLANNING**

- Reinforce use of analgesic
- Reinforce importance of humidification with trach cradle
- Instruct family members on how to best communicate with patient and provide alternate forms of communication for patient if patient unable to speak
- ***No oral suction*** (potentially damages flap and promotes reliance on suction to clear oral secretions)
- Teach importance of swallowing oral secretions (retrains muscles altered by surgery)

Patient Label

***Clinical Care Pathway: Head & Neck Resection and Free Flap Reconstruction***

**POST-OP DAY 3 Date:**

**PATIENT CARE**

- Close Observation Nursing
- **If oral cavity/oropharynx surgery, no oral suction**
- System assessment qshift and prn
- Monitor vital signs q4h and prn
- If JP drain <30cc/24 hours AND not midline, D/C JP
- PT to increase mobilization around ward.
- Change antibiotics from IV to oral/KF
- Trach cuff deflated
- Continuous cold neb
- Maintain SaO2 > 92%
- Chest assessment q4h and prn
- Assess need for tracheal instillations (NS) and suctioning q4h and prn
- Assess tracheal secretions for colour, consistency, volume, and odour
- Deep breathing encouragement/Incentive spirometry
- Trach care with NS and polysporin ointment q4h
- Flap checks q4h. Record each assessment. Report any signs of decreased flap circulation to resident or staff physician immediately
- At each shift change, incoming and outgoing RN to perform combined flap assessment. Both sign assessment form in patient record
- Head of Bed > 30 with head in neutral position
- Encourage patient to swallow own secretions
- Assess for neck edema and symmetry
- Neck incision line care q8h with NS followed by polysporin ointment
- Assess JP output q8h. Reprime and drain q8h and prn
- Reinforce flap donor and skin graft site dressings prn
- Gentle mouth care with NS q4h and prn
- Pain assessment q2h
- D/C Foley if not already done.
- Accurate ins and outs q4h
- Check feeding tube placement qshift
- Ankle flexion and extension exercise q1h during waking hours

**NUTRITION**

- Tube Feeds initiated per RD
- IV TKVO
- If any nausea or vomiting, reconsult dietician
- Flush 250cc H_2_0 via feeding tube q4h

**CONSULTS**

Following:

- Physiotherapy to mobilize around ward.
- Dietician
- Respiratory therapy
- OT

**ACTIVITY**

- Turn and reposition q2h with head neutral
- Activity as tolerated – up to chair bid
- Fibular and ALT free flap – WBAT (no knee flexion until POD#7)

**TEACHING/DISHCARGE PLANNING**

- Reinforce use of analgesic
- Reinforce importance of humidification with trach cradle
- Instruct family members on how to best communicate with patient and provide alternate forms of communication for patient if patient unable to speak
- ***No oral suction*** (potentially damages flap and promotes reliance on suction to clear oral secretions)
- Teach importance of swallowing oral secretions (retrains muscles altered by surgery)

Patient Label

***Clinical Care Pathway: Head & Neck Resection and Free Flap Reconstruction***

**POST-OP DAY 4 Date:**

**PATIENT CARE**

- Close Observation Nursing
- System assessment qshift and prn
- Monitor vital signs q4h and prn
- If JP drain <30cc/24 hours AND not midline, D/C JP
- PT to continue mobilization around ward, increase daily.
- Continue oral antibiotics
- If patient tolerating secretions, able to finger plug and no need for pulmonary toilet, downsize to #4 uncuffed fenestrated Sheiley. If “no” to any, re-assess q24h until all “yes”, then commence plugging trials.
- Continuous cold neb
- Maintain SaO2 > 92%
- Chest assessment q4h and prn
- Assess need for tracheal instillations (NS) and suctioning q4h and prn
- Assess tracheal secretions for colour, consistency, volume, and odour
- Deep breathing encouragement/Incentive spirometry
- Trach care with NS and polysporin ointment q4h
- Flap checks q4h. Record each assessment. Report any signs of decreased flap circulation to resident or staff physician immediately
- At each shift change, incoming and outgoing RN to perform combined flap assessment. Both sign assessment form in patient record
- Head of Bed > 30 with head in neutral position
- Encourage patient to swallow own secretions
- Assess for neck edema and symmetry
- Neck incision line care q8h with NS followed by polysporin ointment
- Assess JP output q8h. Reprime and drain q8h and prn
- Reinforce flap donor and skin graft site dressings prn
- Gentle mouth care with NS q4h and prn
- **If oral cavity/oropharynx surgery, no oral suction**
- Pain assessment q2h
- D/C Foley if not already done.
- Accurate ins and outs q4h
- Check feeding tube placement qshift
- Ankle flexion and extension exercise q1h during waking hours

**NUTRITION**

- IV TKVO
- Tube feeds as ordered.
- If any nausea or vomiting, reconsult dietician
- Flush 250cc H_2_0 via feeding tube q4h

**CONSULTS**

Following:

- Physiotherapy to continue to mobilize around ward.
- Dietician
- Respiratory therapy
- OT

**ACTIVITY**

- Mobilize around ward
- Activity as tolerated – up to chair bid
- Fibular and ALT free flap – WBAT (no knee flexion until POD#7)

**TEACHING/DISHCARGE PLANNING**

- Reinforce use of analgesic
- Reinforce importance of humidification with trach cradle
- Instruct family members on how to best communicate with patient and provide alternate forms of communication for patient if patient unable to speak
- ***No oral suction*** (potentially damages flap and promotes reliance on suction to clear oral secretions)
- Teach importance of swallowing oral secretions (retrains muscles altered by surgery)

Patient Label

***Clinical Care Pathway: Head & Neck Resection and Free Flap Reconstruction***

**POST-OP DAY 5 Date:**

**PATIENT CARE**

- Close Observation Nursing
- System assessment qshift and prn
- Monitor vital signs q4h and prn
- If JP drain <30cc/24 hours AND not midline, D/C JP
- PT to continue mobilization around ward, increase daily.
- Continue oral antibiotics
- If patient tolerating secretions, able to finger plug and no need for pulmonary toilet, downsize to #4 uncuffed fenestrated Sheiley. If “no” to any, re-assess q24h until all “yes”, then commence plugging trials.
- If plugged successfully x24h, decannulate
- Suture stoma when decannulated (stoma sutures to be removed 10 days later)
- SLP to see re: swallowing assessment when decannulated and stoma sutured. If patient fails swallowing assessment, repeat assessment q24hrs AND consider possible PEG tube placement
- Maintain SaO2 > 92%
- Deep breathing encouragement/Incentive spirometry
- Flap checks q4h. Record each assessment. Report any signs of decreased flap circulation to resident or staff physician immediately
- At each shift change, incoming and outgoing RN to perform combined flap assessment. Both sign assessment form in patient record
- Head of Bed > 30 with head in neutral position
- Encourage patient to swallow own secretions
- Assess for neck edema and symmetry
- Neck incision line care q8h with NS followed by polysporin ointment
- Assess JP output q8h. Reprime and drain q8h and prn
- Reinforce flap donor and skin graft site dressings prn
- Gentle mouth care with NS q4h and prn
- **If oral cavity/oropharynx surgery, no oral suction**
- Pain assessment q2h
- D/C Foley if not already done.
- Accurate ins and outs q4h
- Check feeding tube placement qshift
- Ankle flexion and extension exercise q1h during waking hours

**NUTRITION**

- IV TKVO
- Tube feeds as ordered.
- RD to re-assess diet when swallowing.
- If any nausea or vomiting, reconsult dietician
- Flush 250cc H_2_0 via feeding tube q4h

**CONSULTS**

Following:

- Physiotherapy
- Dietician
- Respiratory therapy
- OT
- SLP for swallowing study when decannulated (see SLP care pathway)

**ACTIVITY**

- Mobilize around ward
- Activity as tolerated – up to chair bid
- Fibular and ALT free flap – WBAT (no knee flexion until POD#7)

**TEACHING/DISHCARGE PLANNING**

- Reinforce use of analgesic
- ***No oral suction*** (potentially damages flap and promotes reliance on suction to clear oral secretions)
- Teach importance of swallowing oral secretions (retrains muscles altered by surgery)

Patient Label

***Clinical Care Pathway: Head & Neck Resection and Free Flap Reconstruction***

**POST-OP DAY 6 Date:**

**PATIENT CARE**

- Close Observation Nursing
- System assessment qshift and prn
- Monitor vital signs q4h and prn
- If JP drain <30cc/24 hours AND not midline, D/C JP
- PT to continue mobilization around ward, increase daily.
- Continue oral antibiotics
- If patient tolerating secretions, able to finger plug and no need for pulmonary toilet, downsize to #4 uncuffed fenestrated Sheiley. If “no” to any, re-assess q24h until all “yes”, then commence plugging trials.
- If plugged successfully x24h, decannulate
- Suture stoma when decannulated (stoma sutures to be removed 10 days later)
- SLP to see re: swallowing assessment when decannulated and stoma sutured. If patient fails swallowing assessment, repeat assessment q24hrs AND consider possible PEG tube placement
- Maintain SaO2 > 92%
- Deep breathing encouragement/Incentive spirometry
- Flap checks q4h. Record each assessment. Report any signs of decreased flap circulation to resident or staff physician immediately
- At each shift change, incoming and outgoing RN to perform combined flap assessment. Both sign assessment form in patient record
- Head of Bed > 30 with head in neutral position
- Encourage patient to swallow own secretions
- Assess for neck edema and symmetry
- Neck incision line care q8h with NS followed by polysporin ointment
- Assess JP output q8h. Reprime and drain q8h and prn
- Reinforce flap donor and skin graft site dressings prn
- Gentle mouth care with NS q4h and prn
- **If oral cavity/oropharynx surgery, no oral suction**
- Pain assessment q2h
- D/C Foley if not already done.
- Accurate ins and outs q4h
- Check feeding tube placement qshift
- Ankle flexion and extension exercise q1h during waking hours

**NUTRITION**

- IV TKVO
- Tube feeds as ordered.
- RD to re-assess diet when swallowing.
- If any nausea or vomiting, reconsult dietician
- Flush 250cc H_2_0 via feeding tube q4h

**CONSULTS**

Following:

- Physiotherapy
- Dietician
- Respiratory therapy
- OT
- SLP

**ACTIVITY**

- Mobilize around ward
- Activity as tolerated – up to chair bid
- Fibular and ALT free flap – WBAT (no knee flexion until POD#7)

**TEACHING/DISHCARGE PLANNING**

- Reinforce use of analgesic
- ***No oral suction*** (potentially damages flap and promotes reliance on suction to clear oral secretions)
- Teach importance of swallowing oral secretions (retrains muscles altered by surgery)

Patient Label

***Clinical Care Pathway: Head & Neck Resection and Free Flap Reconstruction***

**POST-OP DAY 7 Date:**

**PATIENT CARE**

- Close Observation Nursing
- System assessment qshift and prn
- Monitor vital signs q4h and prn
- If JP drain <30cc/24 hours AND not midline, D/C JP
- PT to continue mobilization around ward, increase daily.
- Continue oral antibiotics
- If patient tolerating secretions, able to finger plug and no need for pulmonary toilet, downsize to #4 uncuffed fenestrated Sheiley. If “no” to any, re-assess q24h until all “yes”, then commence plugging trials.
- If plugged successfully x24h, decannulate
- Suture stoma when decannulated (stoma sutures to be removed 10 days later)
- SLP to see re: swallowing assessment when decannulated and stoma sutured. If patient fails swallowing assessment, repeat assessment q24hrs AND consider possible PEG tube placement
- Dressings down
- Skin graft donor site – xeroform to air (trim prn)
- Free flap donor site: If >90% skin graft take, adaptic 🡪 dry gauze 🡪 kling.
- If <90% and >50% graft take, polysporin 🡪 Dry gauze 🡪 kling
- If <50% graft take, saline soaked cotton gauze, wet to dry, BID
- If no previous XRT, barium swallow to r/o anastomotic leak
- Maintain SaO2 > 92%
- Deep breathing encouragement/Incentive spirometry
- Flap checks q4h. Record each assessment. Report any signs of decreased flap circulation to resident or staff physician immediately
- At each shift change, incoming and outgoing RN to perform combined flap assessment. Both sign assessment form in patient record
- Head of Bed > 30 with head in neutral position
- Encourage patient to swallow own secretions
- Assess for neck edema and symmetry
- Neck incision line care q8h with NS followed by polysporin ointment
- Assess JP output q8h. Reprime and drain q8h and prn
- Reinforce flap donor and skin graft site dressings prn
- Gentle mouth care with NS q4h and prn
- **If oral cavity/oropharynx surgery, no oral suction**
- Pain assessment q2h
- D/C Foley if not already done.
- Accurate ins and outs q4h
- Check feeding tube placement qshift
- Ankle flexion and extension exercise q1h during waking hours

**NUTRITION**

- IV TKVO
- Tube feeds as ordered.
- RD to re-assess diet when swallowing.
- Diet as ordered if swallowing
- If any nausea or vomiting, reconsult dietician
- Flush 250cc H_2_0 via feeding tube q4h

**CONSULTS**

Following:

- Physiotherapy
- Dietician
- Respiratory therapy
- OT
- SLP

**ACTIVITY**

- Mobilize around ward
- Activity as tolerated – up to chair bid
- Fibular and ALT free flap – WBAT gentle knee flexion initiated.

**TEACHING/DISHCARGE PLANNING**

- Reinforce use of analgesic
- ***No oral suction*** (potentially damages flap and promotes reliance on suction to clear oral secretions)
- Teach importance of swallowing oral secretions (retrains muscles altered by surgery)

Patient Label

***Clinical Care Pathway: Head & Neck Resection and Free Flap Reconstruction***

**POST-OP DAY 8 Date:**

**PATIENT CARE**

- Close Observation Nursing
- System assessment qshift and prn
- Monitor vital signs q4h and prn
- If JP drain <30cc/24 hours AND not midline, D/C JP
- PT to continue mobilization around ward, increase daily.
- ADL assessment
- Continue oral antibiotics
- If not decannulated, discuss PEG placement
- SLP to see re: swallowing assessment when decannulated and stoma sutured. If patient fails swallowing assessment, repeat assessment q24hrs AND consider possible PEG tube placement
- Continue dressing changes as ordered
- If no previous XRT, barium swallow to r/o anastomotic leak
- Maintain SaO2 > 92%
- Deep breathing encouragement/Incentive spirometry
- Flap checks q8h. Record each assessment. Report any signs of decreased flap circulation to resident or staff physician immediately
- At each shift change, incoming and outgoing RN to perform combined flap assessment. Both sign assessment form in patient record
- Head of Bed > 30 with head in neutral position
- Encourage patient to swallow own secretions
- Assess for neck edema and symmetry
- Neck incision line care q8h with NS followed by polysporin ointment
- Assess JP output q8h. Reprime and drain q8h and prn
- Reinforce flap donor and skin graft site dressings prn
- Gentle mouth care with NS q4h and prn
- **If oral cavity/oropharynx surgery, no oral suction**
- Pain assessment q2h
- Accurate ins and outs q4h

**NUTRITION**

- IV TKVO
- Tube feeds as ordered.
- RD to re-assess diet when swallowing.
- Diet as ordered if swallowing
- If any nausea or vomiting, reconsult dietician
- Flush 250cc H_2_0 via feeding tube q4h

**CONSULTS**

**Following:**

- Physiotherapy
- Dietician
- Respiratory therapy
- OT
- SLP
- GI/Radiology/Gen Sx for G-tube placement if needed

**ACTIVITY**

- Mobilize around ward
- Fibular and ALT free flap – WBAT gentle knee flexion okay.

**TEACHING/DISHCARGE PLANNING**

- Reinforce use of analgesic
- Set-up home care
- CCI referral
- Trach teaching if trach still in place
- G-tube teaching if G-tube placed
- ***No oral suction*** (potentially damages flap and promotes reliance on suction to clear oral secretions)
- Teach importance of swallowing oral secretions (retrains muscles altered by surgery)

Patient Label

***Clinical Care Pathway: Head & Neck Resection and Free Flap Reconstruction***

**POST-OP DAY 9 Date:**

**PATIENT CARE**

- Close Observation Nursing
- System assessment/vitals qshift and prn
- D/C midline JP
- PT to continue mobilization around ward, increase daily.
- D/C dopplers
- Last dose of oral antibiotics
- Continue dressing changes as ordered
- D/C continuous O2 Sat monitoring (if safe to do so)
- Deep breathing encouragement/Incentive spirometry
- Neck incision line care q8h with NS followed by polysporin ointment
- Reinforce flap donor and skin graft site dressings prn
- **If oral cavity/oropharynx surgery, no oral suction**
- Pain assessment q2h
- D/C home if:
- Stable
- Home-care set up
- CCI appointment arranged
- Swallowing well, OR G-tube placed and patient G-tube teaching completed
- Adequate po pain control achieved
- SLP followup arranged PRN
- IRSM followup appointments made
- OT to D/C splints in RFFF and FFF patients
- D/C IVs

**NUTRITION**

- IV TKVO
- Tube feeds as ordered.
- RD to re-assess diet when swallowing.
- Diet as ordered if swallowing
- If any nausea or vomiting, reconsult dietician
- Flush 250cc H_2_0 via feeding tube q4h

**CONSULTS**

**Following:**

- Physiotherapy
- Dietician
- Respiratory therapy
- OT
- SLP
- GI/Radiology/Gen Sx for G-tube placement if needed

**ACTIVITY**

- Mobilize around ward
- Fibular and ALT free flap – WBAT gentle knee flexion okay.

**TEACHING/DISHCARGE PLANNING**

- Reinforce use of analgesic
- Set-up home care
- CCI referral
- Trach teaching if trach still in place
- G-tube teaching if G-tube placed
- ***No oral suction*** (potentially damages flap and promotes reliance on suction to clear oral secretions)
- Teach importance of swallowing oral secretions (retrains muscles altered by surgery)
