# Supplementary material for: Algorithm based patient care protocol to optimize patient care and inpatient stay in head and neck free flap patients
Source: J Otolaryngol Head Neck Surg. 2015 Nov 2;44:45. doi: 10.1186/s40463-015-0090-6 (PMC4631082; doi:10.1186/s40463-015-0090-6)
Supplement: Additional file 2: — Head and neck reconstruction flow sheet. Daily progress note template. (PDF 1004 kb) [file 40463_2015_90_MOESM2_ESM.pdf]

Alberta Health Services

Affix patient label within this box.

Head and Neck Reconstruction Flow Sheet

Post-Operative Day	**Date** *(yyyy-Mon-dd)*

Flap type
☐ RFFF ☐ ALT ☐ Fibula ☐ Other *(specify)* ______

**Trach size**	**Cuff-up** ☐ Yes ☐ No	**Decannulated** ☐ Yes ☐ No
**Trach secretions**<br>☐ Mild<br>☐ Moderate<br>☐ Copious	☐ SaO2 ______ %<br>☐ Room Air<br>☐ ______ L	**Are vital signs stable?**<br>☐ Yes<br>☐ No, abnormals ______

**Dopplers**	**Incisions**	**JP #**	**cc/24 hr**	**d/c'd (✔)**
**Arterial**<br>☐ Strong ☐ Concern<br>**Venous**<br>☐ Strong ☐ Concern<br>Comments ______	**Flap – Recon**<br>☐ Intact ☐ Dehiscence<br>**Donor-Site**<br>☐ Intact ☐ Dehiscence<br>**Neck**<br>☐ Intact ☐ Dehiscence<br>Comments ______			

Activity
☐ Bed Rest ☐ Up to Chair ☐ Mobilizing with assist ☐ AAT

Diet
☐ Tube feeds ☐ Continuous *(specify)* rate ______ cc/hr ☐ Bolus

Has patient had a bowel movement? ☐ Yes ☐ No

Swallow assessment ☐ Pass ☐ Fail

PO Diet	Calorie Counts ☐ 25% ☐ 50% ☐ 75% ☐ 100%

Labs

Hgb	WBC	iCa+	Other *(specify)*

Is protocol being followed? ☐ Yes ☐ No

Comments/Other Issues

______

Notes

______

18031(2011-06)
