# Supplementary material for: Algorithm based patient care protocol to optimize patient care and inpatient stay in head and neck free flap patients
Source: J Otolaryngol Head Neck Surg. 2015 Nov 2;44:45. doi: 10.1186/s40463-015-0090-6 (PMC4631082; doi:10.1186/s40463-015-0090-6)
Supplement: Additional file 3: — Head and neck reconstruction intake form. Intake form for head and neck reconstruction patients. (PDF 1015 kb) [file 40463_2015_90_MOESM3_ESM.pdf]

Alberta Health Services

Affix patient label within this box

Head and Neck Reconstruction Intake

Diagnosis

T	N	M	☐ Squamous Cell Carcinoma<br>☐ Other *(specify)* ______

Site

☐ Right ☐ Left ☐ Midline

☐ **Oral cavity**	☐ **Oropharynx**	☐ **Larynx**
☐ Tongue ☐ FOM<br>☐ Mandible ☐ Hard Palate<br>☐ RMT ☐ Buccal<br>☐ Lip	☐ BOT<br>☐ Tonsil<br>☐ SP	☐ Supraglottic<br>☐ Glottic<br>☐ Subglottic

☐ **Other** *(specify)* ______

Past Medical History

☐ Smoker<br>☐ DM2<br>☐ CAD	☐ IDDM<br>☐ A-Fib<br>☐ Prev MI	☐ Anticoags pre-op<br>☐ ASA<br>☐ Coumadin<br>☐ LMWH	☐ EtOH<br>Risk of withdrawl?<br>☐ Yes<br>☐ No

Surgery Resection

Approach ☐ Transoral ☐ Transmandibular ☐ Total Laryngectomy ☐ Other*(specify)* ______

Neck Dissections

**Right**	☐ MRND	☐ SND *(specify)*	☐ I	☐ IIA	☐ IIB	☐ III	☐ IV	☐ V
**Left**	☐ MRND	☐ SND *(specify)*	☐ I	☐ IIA	☐ IIB	☐ III	☐ IV	☐ V

Surgery Defect

Skin

Mucosa

Bone

Nerves ☐ cut, describe ______

Nerve repair ☐ primary ☐ cablegraft ☐ flap innervated

Suprahyoid muscle ☐ intact ☐ resected ( ) | Mylohyoid nerve ☐ intact ☐ cut

Surgery Reconstruction

			Modification
☐ Right<br>☐ Left	☐ RFFF<br>☐ ALT	☐ Fibula<br>☐ Other *(specify)* ______	☐ SPIR<br>☐ BIFF<br>☐ Beavertail
☐ Right<br>☐ Left	☐ RFFF<br>☐ ALT	☐ Fibula<br>☐ Other *(specify)* ______	

Arterial Anas No.1 ______ Venous Anas No. 1 ______

Arterial Anas No. 2 ______ Venous Anas No. 2 ______

Tourniquet time flap # 1 *(hh:mm)*	Tourniquet time flap # 2 *(hh:mm)*	Vessel clamp *(hh:mm)*	Vessel flow *(hh:mm)*
		Vessel clamp *(hh:mm)*	Vessel flow *(hh:mm)*

Hgb	Transfusion	Fluids	Inotropes intra-op
pre ______<br>post ______	☐ Yes *(specify)* ______ units pRBCs<br>☐ No	■ Crystalloid ______ cc<br>■ Colloid ______ cc	☐ Yes<br>☐ No

Comments

18030(Rev2011-10)
